# Anticoagulant SERPINs: Endogenous Regulators of Hemostasis and Thrombosis

**DOI:** 10.3389/fcvm.2022.878199

**Published:** 2022-05-03

**Authors:** Steven P. Grover, Nigel Mackman

**Affiliations:** Division of Hematology and Oncology, Department of Medicine, UNC Blood Research Center, University of North Carolina at Chapel Hill, Chapel Hill, NC, United States

**Keywords:** anticoagulants, coagulation factors, hemostasis, serine protease inhibitors, thrombosis

## Abstract

Appropriate activation of coagulation requires a balance between procoagulant and anticoagulant proteins in blood. Loss in this balance leads to hemorrhage and thrombosis. A number of endogenous anticoagulant proteins, such as antithrombin and heparin cofactor II, are members of the serine protease inhibitor (SERPIN) family. These SERPIN anticoagulants function by forming irreversible inhibitory complexes with target coagulation proteases. Mutations in SERPIN family members, such as antithrombin, can cause hereditary thrombophilias. In addition, low plasma levels of SERPINs have been associated with an increased risk of thrombosis. Here, we review the biological activities of the different anticoagulant SERPINs. We further consider the clinical consequences of SERPIN deficiencies and insights gained from preclinical disease models. Finally, we discuss the potential utility of engineered SERPINs as novel therapies for the treatment of thrombotic pathologies.

## Introduction

**FIGURE 1:**
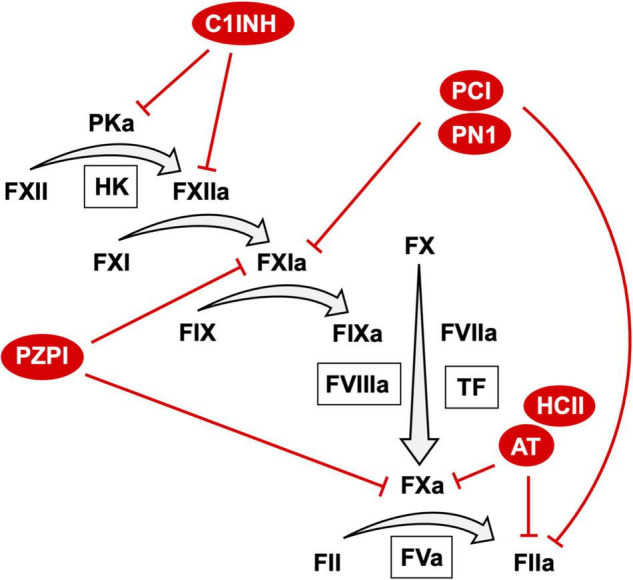
The coagulation cascade and anticoagulant SERPIN targets. A schematic representation of the coagulation cascade annotated with anticoagulant SEPRIN targets. For clarity only the top targets of each SERPIN with second order inhibitory rate constants > 1.0 × 10^5^ are represented. A comprehensive list of targets and second order inhibitory rate constants is provided in Table 1.

Appropriate activation of coagulation is essential in limiting blood loss from a closed circulatory system. On vascular injury, exposure of sub-endothelial tissue factor (TF) results in activation of the extrinsic pathway of coagulation through exposure of the TF: factor (F) VIIa complex to substrate FX resulting in generation of FXa (Figure 1) (1). Coagulation can also be initiated by activation of the intrinsic pathway through autoactivation of FXII to FXIIa (2, 3). FXIIa generation is enhanced by reciprocal activation of FXII by plasma kallikrein and its cofactor high molecular weight kininogen. FXIIa activates FXI to FXIa that itself activates FIX to FIXa. FIXa in complex with cofactor FVIIIa catalyzes additional FXa generation (Figure 1). FXa, in complex with the essential cofactor FVa and in the presence of additional cofactors phospholipid and calcium, catalyzes the conversion of prothrombin zymogen to thrombin. As the terminal coagulation protease thrombin catalyzes cleavage of soluble fibrinogen to insoluble fibrin leading to the formation of a fibrin mesh that functions to limit blood loss at the site of injury. Additionally, thrombin facilitates the formation of a platelet rich plug at the site of vascular injury through direct activation of platelets.

Thrombin generation can be further enhanced through feedback activation of the intrinsic pathway of coagulation by the extrinsic pathway. The TF:FVIIa complex is an effective activator of FIX and the TF:FVIIa:FXa ternary complex can activate FVIII (4, 5). In addition, thrombin can directly activate FXI leading to further intrinsic pathway derived thrombin generation (6). Together these feedback pathways can generate significant quantities of thrombin and contribute to hemostasis in a tissue specific manner (7).

Inappropriate activation of coagulation underpins a number of common cardiovascular and hematological diseases. An impaired ability to mount an effective hemostatic response to vascular injury can lead to bleeding disorders, such as those observed in individuals with genetic deficiencies for FVIII and FIX that cause hemophilia A and B, respectively (8). Alternatively, excessive activation of coagulation can lead to thrombotic disorders such as venous thromboembolism (VTE) that includes both deep vein thrombosis (DVT) and pulmonary embolism (PE) (1, 2, 9). Excessive activation of coagulation can be caused by resistance of coagulation factors to inhibition, such as is observed in Factor V Leiden, (10).

For this reason, the coagulation system must be tightly regulated. Negative regulation of coagulation is achieved by anticoagulant proteins. These anticoagulant proteins take the form of protease inhibitors that together function to inactivate all proteases of the coagulation system. A number of these protease inhibitors are members of the serine protease inhibitor (SERPINs) superfamily (11). Anticoagulant SERPINs include antithrombin (AT), heparin co-factor II (HCII), protein Z dependent protease inhibitor (PZPI), protease nexin 1 (PN1) and C1-inhibitor (C1INH) (Figure 1). The inhibitory activity of SERPINs is complemented by a number of non-SERPIN anticoagulants. This includes tissue factor pathway inhibitor, which is the primary inhibitor of the TF:FVIIa complex (12). In addition, activated protein C (aPC) and its essential cofactor protein S function as an important physiological inhibitor of FVa and FVIIIa (13).

In this review, we introduce the properties of SERPIN family members that facilitate inhibition of target coagulation proteases. We further discuss the anticoagulant properties of specific SERPINs and consider how these properties enable regulation of hemostatic and thrombotic processes. Particular attention is paid to congenital SERPIN deficiencies, more broadly referred to as serpinopathies, that are associated with altered thrombotic risk (14, 15). Finally, we review advances in the development of variant SERPIN proteins with therapeutic potential.

## Structure and Function of Anticoagulant Serine Protease Inhibitor

### Nomenclature

Despite the identification of numerous non-inhibitory family members, the SERPIN superfamily name remains. Members are subdivided into clades based on phylogenetics. To date, sixteen clades, named A through P, have been identified with an additional number of SERPINs remaining as unclassified orphan members (16, 17). Based on the clade naming system, alpha 1 antitrypsin (A1AT) is the first member of clade A and is designated SERPINA1 encoded by the *SERPINA1* gene (16, 17). Despite the unifying clade-based nomenclature for SERPINs the majority of family member proteins are referred to by the name given to them on first description. SERPINs with anticoagulant activities are dispersed throughout the clades (Table 1).

**TABLE 1:** Anticoagulant SERPINs and targets.

Protein	Gene	Molecular weight (kDa)	Coagulation targets	Rate constant (M–1 s–1)	References	Non-coagulation targets	Rate constant (M–1 s–1)	References
A1AT	*SERPINA1*	53	FXa FXIa	1.3 × 10^2^ 1.0 × 10^2^	(207) (37)	NE PR3 CG	1.3 × 10^7^ 8.1 × 10^6^ 4.1 × 10^5^	(208) (206) (204)
PCI	*SERPINA5*	46	FIIa aPC FXIa PKa FXa	2.4 × 10^6 tm^ 1.5 × 10^6 h^ 7.4 × 10^5 h^ 1.8 × 10^5 h^ 9.0 × 10^4 h^	(192) (191) (191) (191) (191)			
PZPI	*SERPINA10*	72	FXIa FIXa FXa	7.7 × 10^5 h^ 5.4 × 10^5^ 3.6 × 10^5 h^	(114) (116) (114)			
AT	*SERPINC1*	58	FIIa FXa FIXa PKa FXIa	6.1 × 10^7 h^ 4.4 × 10^7 h^ 9.0 × 10^6 h^ 1.9 × 10^5 h^ 1.5 × 10^3 ds^	(41) (41) (266) (267) (37)			
HCII	*SERPIND1*	65	FIIa FXa	4.5 × 10^8 h^ 3.0 × 10^5 h^	(83) (83)			
PN1	*SERPINE2*	50	FIIa FXIa FXa	1.2 × 10^9 h^ 1.7 × 10^6 h^ 3.5 × 10^5 h^	(173) (174) (173)	Trypsin uPA Plasmin	1.0 × 10^7 h^ 9.6 × 10^5^ 1.0 × 10^5 h^	(173) (173) (173)
C1INH	*SERPING1*	105	PKa FXIIa FXIa FIIa	2.9 × 10^6^ 4.3 × 10^5^ 2.1 × 10^5 ds^ 1.3 × 10^4^	(268) (269) (37) (270)	C1r C1s Plasmin tPA	NR 3.4 × 10^5^ NR NR	(244)

*ds, in the presence of dermatan sulfate; h, in the presence of heparin; tm, in the presence of thrombomodulin. NR, not reported.*

### Protein Structure

**FIGURE 2:**
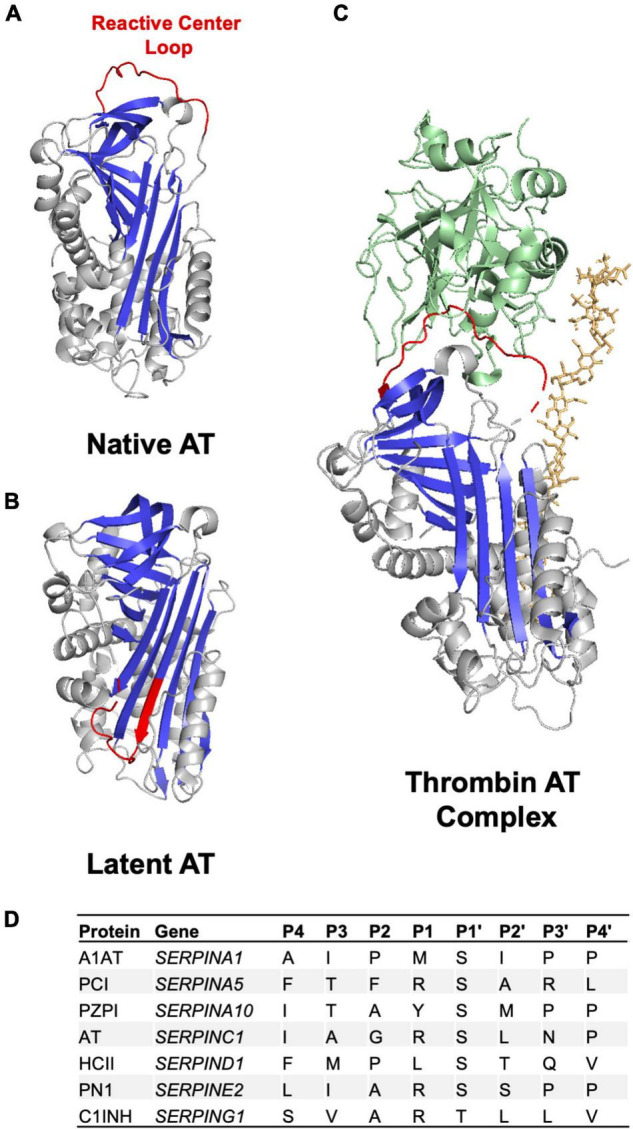
Anticoagulant SERPIN structure and RCL. **(A)** Crystal structure of the anticoagulant SERPIN AT in the native conformation with a protease accessible RCL **(B)** Crystal structure of AT in the latent conformation in which the RCL is buried in the protein body and inaccessible to protease. The AT RCL is colored red, β sheets are colored in blue and α helices in gray. **(C)** Crystal structure of AT in complex with thrombin (green) in the presence of heparin (orange). Images made in PyMol using PDB files 1T1F, 2BEH, and 1TB6. **(D)** Amino acid residues in anticoagulant SERPINs in the P4-P4’ region of the RCL.

The initial identification of the SERPIN family of serine protease inhibitors was based on primary sequence similarities identified between AT and A1AT (18). Subsequent structural studies demonstrated that, despite relatively low sequence identity, relatively high levels of sequence homology enabled SERPINs to retain a well-preserved sequence of secondary structures consisting of three β-sheets and 8–9 α-helices that form a core structural domain (Figure 2) (16). In addition, SERPINs contain a reactive center loop (RCL) that functions as a critical determinant for protease specificity (19). As a result of these structural similarities the SERPIN family of proteins demonstrate a remarkable degree of structural homology (20). Outside of this core structural domain SERPINs possess variable N and C terminal regions that contribute to the wide range of observed molecular weights. For example, unlike the smaller SERPINs A1AT and PCI that are 45–55 kDa in weight, C1INH inhibitor possesses a heavily glycosylated N-terminal extension resulting in an observed molecular weight of 105 kDa.

### The Reactive Center Loop

The RCL is a 16–17 amino acid sequence found toward the SERPIN C terminus (Figure 2) (21). This sequence is critical to the inhibitory specificity of a given SERPIN. The RCL contains a protease recognition sequence that functions as molecular “bait” for target proteases (19). A target protease binds to the SERPIN through the protease recognition sequence and forms a reversible Michaelis-Menten complex. The protease recognition sequence contains a proteolytic cleavage site with RCL amino acid residues being annotated with P for N terminal residues to the cleavage site and P’ for C terminal residues to the cleavage site as per convention (Figure 2) (22). The specificity of a given SERPIN for a given protease appears to be particularly dependent on the amino acids present at the P1-P1’ positions but can also be influenced by the sequence of amino acids in the P4-P4’ positions (Figure 2) (19). Cleavage of the SERPIN by a target protease results in the formation of a covalent bond between the protease and the main chain carbonyl bond of the SERPIN P1 residue (21). This SERPIN cleavage event results in a marked conformational change as discussed below.

### Conformational Change

SERPINs can exist in either a native metastable or a non-native hyperstable conformational state (Figure 2). In the native metastable conformation, the RCL is externalized and free to interact with target proteases (21). Upon cleavage by a target protease the RCL inserts into β-sheet A forming the hyperstable conformation. Insertion of the RCL into the SERPIN body results in translocation of the target protease interrupting the final hydrolysis of the bond between SERPIN and protease. Trapping of the protease in this manner prevents release and results in the formation of an irreversible SERPIN-protease complex (21). The speed with which a SERPIN, when cleaved by a protease, transitions between the native metastable state and latent hyperstable state is important in determining inhibitory capacity (19, 21). A slow transition allows for hydrolysis of the bond between the SERPIN and protease regenerating functional protease and leaving hyperstable SERPIN. In addition to inhibiting the proteolytic function of the protease, SERPIN complex formation also significantly disrupts the structure of the protease making it more susceptible to proteolytic degradation (23).

It is also possible for SERPINs to be present in a latent conformational state. The latent conformational state bears some structural similarity to the hyperstable state in that the RCL inserts into β-sheet A but this process is not dependent on interaction with a target protease (21). Interestingly, AT has been found to be present as a conformational heterodimer with one monomer present in the latent state (24).

### Glycosaminoglycan Based Activation

**FIGURE 3:**
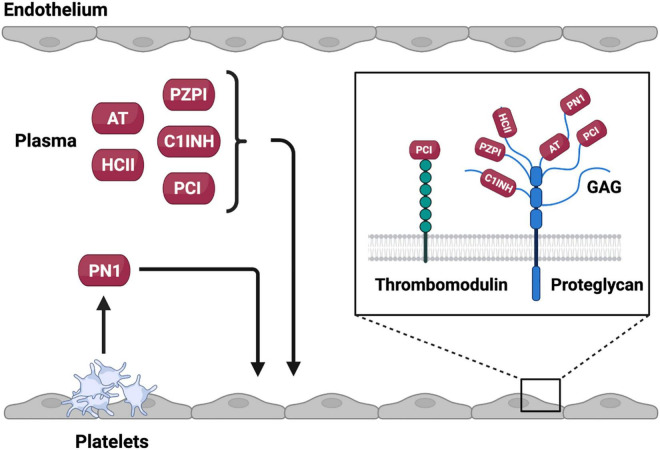
Source and site of activity of anticoagulant SERPINs. Anticoagulant SERPINs AT, HCII, PZPI, C1INH, and PCI are present in the plasma whereas PN1 is stored in platelet alpha granules where it can be released on platelet activation. Anticoagulant SERPINs bind to glycosaminoglycans (GAGs) or protein cofactors on the endothelial surface that potentiate inhibition of target proteases. Created with BioRender.com.

The inhibitory capacity of most anticoagulant SERPINs, including AT, C1INH, HCII PCI, and PN1, is enhanced by binding to negatively-charged glycosaminoglycans (GAGs), such as heparin (Figure 3) (25). The inhibitory activity of these SERPINs can be increased by as much as several thousand-fold by GAG binding. The ability of the GAG heparin to potentiate the activity of anticoagulant SERPINs has been leveraged clinically with unfractionated and low molecular weight formulations used as thromboprophylactic agents to prevent VTE (26).

Two major mechanisms of GAG mediated rate enhancement have been proposed. First, GAGs can act as a bridge binding to both the SERPIN and the protease bringing them together in a confirmation preferable for the protease to interact with the RCL. This interaction has been described to occur between AT and thrombin in the presence of heparin (Figure 2) (27, 28). Second, binding of the GAGs to the SERPIN, which may occur at a distal site, can result in a conformational change in the SERPIN that makes it more reactive toward the target protease. GAGs have been found to facilitate this interaction between HCII and thrombin (29–31). It is likely that GAGs function as both bridging molecules and allosteric modulators for a given SERPIN (32). Indeed, the native forms of both AT and HCII have RCLs that are partially incorporated into the SERPIN body with GAG binding promoting RCL exposure (31, 33, 34). GAGs are present on the endothelial surface forming part of the glycocalyx. Binding of SERPINs to GAGs facilitates localization of these inhibitors to the vessel surface and may help prevent inappropriate intravascular coagulation. At sites of vessel injury where endothelial bound GAGs are not present activation of coagulation is allowed to continue to form a hemostatic plug. This property of SERPINs may be of particular interest when considering the use of these inhibitors as therapeutic agents.

## Antithrombin

### Biological Properties

AT is a 58 kDa serine protease inhibitor encoded by the *SERPINC1* gene. AT is present in plasma at a concentration of approximately 120 μg/ml with a circulating half-life of roughly 3 days. AT functions as a major endogenous inhibitor of thrombin and FXa but also inhibits FIXa, FXIa, FXIIa, and plasma kallikrein (Table 1) (35–41). The ability of AT to inhibit target proteases is dramatically enhanced (2,000–4,000 times) in the presence of heparin (42–44). Interestingly, the GAG heparan sulfate appears to be a much more effective potentiator of AT activity than others, such as dermatan sulfate and chondroitin sulfate (45). This is likely due to the fact that heparin and heparan sulfate contain a specific sulfated pentasaccharide (46–48). The RCL of native AT was shown to be partially incorporated into the SERPIN body limiting interaction with target proteases (33, 34). Critically, binding of the specific heparin pentasaccharide induced a confirmational change in native AT expelling the RCL from the SERPIN body providing accessibility to target proteases (49, 50). The altered presentation of the AT reactive center loop may underpin the enhanced inhibitory activity observed in the presence of heparin. As discussed earlier native and latent AT can form heterodimers (24, 33). Formation of heterodimers was found to reduce the thrombin inhibitory capacity of AT (24). It has been proposed that some AT variants associated with VTE, that retain significant activity against thrombin, may have an increased tendency to be present in the latent conformation and may disrupt the inhibitory capacity of native AT through heterodimer formation (24, 51, 52).

### Clinical Phenotypes

Congenital deficiency for AT is relatively common in the general population with an estimated prevalence of between 1:500 and 1:2,000 (53, 54). To date more than 200 mutations in the *SERPINC1* gene have been identified that cause AT deficiency (55). Mutations in the *SERPINC1* gene can lead to reduced levels of AT, causing type I AT deficiency, or to expression of AT with reduced anticoagulant activity, causing type II AT deficiency (56). Type II AT deficiency can be further divided into additional subtypes: type IIa caused by mutations in the thrombin binding site, type IIb caused by mutations in the heparin binding site or type IIc in which mutations near the reactive center loop have pleiotropic effects on both heparin and thrombin interactions (56). Interestingly, a *SERPINC1* variant causing type II AT deficiency has been shown to induce polymerization of AT in the plasma (57). This mechanism is distinct from other SERPIN family variants described that destabilize protein structure and cause intracellular aggregation and retention. Type II AT deficiencies are much more common in the general population than type I deficiencies (58).

Between 1 and 2% of all VTE events are associated with AT deficiency (59, 60). Strikingly, individuals with AT deficiency have a 50% chance of suffering a VTE by the age of 50 (61, 62). Consistent with this, individuals with AT deficiency have a markedly increased risk of a first VTE compared to controls (relative risk 8.1) (Table 2) (61). Further, a number of studies have reported an increase in the rate of VTE recurrence in individuals with overt (< 70% normal AT levels) deficiency (hazard ratios 1.9–5.9) (63–65). On presenting with a first proximal DVT event individuals with AT deficiency had an increased risk of symptomatic PE (relative risk 2.4) (60). Interestingly, individuals with mild AT deficiency (70–80% of normal) also have an increased risk of venous thromboembolism recurrence (hazard ratio 3.9) (64). The robustness of this observation is unclear, however, with a subsequent study finding a weaker not significant association (65). AT deficiency appears to be associated with a modestly increased risk of arterial thrombosis (66, 67). Further, low plasma levels of AT were found to be associated with a significantly increased risk of arterial thrombotic events (68). Additional studies are warranted to determine the effect of AT deficiency on risk of arterial thrombosis. Importantly, human purified plasma-derived AT products with elimination half-lives of 2–3 days are used for thromboprophylactic replacement therapy in patients with AT deficiency undergoing surgical procedures (69).

**TABLE 2:** Associations between SERPIN abnormalities and atherothrombotic diseases.

Condition	Disease association	Potential therapies	References
AT deficiency	↑ VTE	AT replacement	(59–65)
HCII deficiency	↑ Atherosclerosis	-	(96–100)
PZPI deficiency	↑ VTE	-	(118–120)
Low plasma C1INH	↑ VTE	C1INH replacement	(165)
A1AT deficiency	↑ VTE	A1AT replacement	(219, 220)
A1AT Pittsburgh	↑ Bleeding		(222, 226)

AT is a negative acute phase reactant, being depleted under inflammatory conditions, that is also thought to have important anti-inflammatory functions (70). In the inflammatory setting of sepsis patients that did not survive had significantly lower plasma levels of AT than those that survived (71). Consistent with the anti-inflammatory effects of AT, treatment of sepsis patients with exogenous AT significantly reduced plasma levels of interleukin 6 and c reactive protein (72). Given the proinflammatory activities of AT targets FXa and thrombin it is likely that the anti-inflammatory effects of AT are secondary to inhibition of FXa (70).

### Preclinical Phenotypes

Attempts have been made to generate AT deficient mice (73). Complete deficiency for AT was found to result in embryonic lethality at mid-gestation (73). AT deficient embryos had evidence of intravascular fibrin deposition and extensive hemorrhage consistent with a consumptive coagulopathy (73). Interestingly, transgenic mice expressing an AT variant, R48C, with reduced ability to bind heparin cofactor have also been generated (74). Embryos homozygous for this variant AT were present at mid-gestation at the expected frequency (74). However, reduced peri and post-natal survival was observed with mice homozygous for the variant AT (74). In addition, adult mice homozygous for the variant AT had evidence of spontaneous thrombosis, particularly in the heart and liver (74). In a complementary approach short interfering RNA mediated silencing of the *SERPINC1* gene in mice resulted in acute and extensive thrombus formation in the head, limbs and liver (75). Crossing of AT deficient mice with those expressing low levels of TF enabled deficient embryos to persist to late gestation but was not sufficient to generate viable mice (76). Disruption of AT in zebrafish also resulted in reduced survival of AT deficient offspring with evidence of spontaneous thrombosis (77). Surprisingly, AT deficient larvae demonstrated prolonged occlusion times in a caudal vein laser injury thrombosis model (77). This was explained by the presence of hypofibrinogenemia in AT deficient larvae likely caused by a consumptive coagulopathy (77). These studies demonstrate that the anticoagulant activity of AT is essential for appropriate regulation of coagulation *in vivo*. Further, loss of AT anticoagulant activity can lead to spontaneous thrombotic events.

In light of the critical anticoagulant activity of AT, inhibition of this protein has been explored as a potential therapy for hemophilia (78). FVIII deficient mice that were also heterozygous for AT had increased thrombin generation and demonstrated shortened bleeding times in a tail transection model compared to FVIII deficient mice (79). Short interfering RNA mediated gene silencing of the *SERPINC1* gene was also found to reduce bleeding in FVIII deficient mice subject to a saphenous vein transection model (80). *SERPINC1* gene silencing in FVIII deficient mice supported increased platelet and fibrin accumulation at sites of vascular injury in a cremaster arteriole laser injury model of thrombosis (80). A single-domain inhibitory anti-AT antibody has also been developed (81). FVIII or FIX deficient mice administered an adenoviral vector expressing the single-domain inhibitory anti-AT antibody had significantly reduced blood loss in a tail vein transection bleeding model compared to controls (81). Importantly, this anti-AT antibody was equally as effective in reducing blood loss in FIX deficient mice with inhibitory FIX autoantibodies compared to those without (81). Based on these studies, a *SERPINC1* targeting short interfering RNA is currently being evaluated in patients with hemophilia A and B (82).

### Summary

AT functions as a potent inhibitor of thrombin and FXa. A significant body of clinical evidence indicates that AT deficiency is associated with an increased risk of VTE. Consistent with the essential anticoagulant functions of AT mice deficient for this SERPIN are not viable. AT inhibitors have shown promise as prohemostatic therapies in preclinical models of hemophilia supporting enhanced thrombin generation.

## Heparin Cofactor II

### Biological Properties

HCII, encoded by the *SERPIND1* gene, is a 65 kDa serine protease inhibitor that serves as a potent and selective inhibitor of thrombin and to a lesser extent FXa (Table 1) (83). HCII is present in plasma at a concentration of approximately 80μg/ml with a circulating half-life of 2–3 days (83, 84). Unlike AT, HCII is a stronger inhibitor of thrombin and has markedly weaker activity against FXa (83). As is the case with AT, native HCII is a poor inhibitor of thrombin owing to the lack of accessibility of the RCL in this conformation (31). Binding of HCII to GAGs drives the conformational transformation of native inactive HCII into the native active form expelling the partially incorporated RCL and markedly enhancing activity toward target proteases (31). Unlike AT, that is selectively activated by specific pentasaccharides present in a small percentage of GAGs, HCII is less selective and can be activated by a host of GAGs, including heparan sulfate, chondroitin sulfate, and dermatan sulfate (85–87).

### Clinical Phenotypes

HCII deficiency in humans has been described, with a number of individuals identified after episodes of arterial or venous thrombosis (88–92). In two studies patients with a history of arterial and venous thrombosis were found to have plasma HCII levels 50% of normal (88, 89). Further, an individual presenting with multiple episodes of DVT and PE was found to have a homozygous deficiency for HCII (91). This individual was also found to have a heterozygous AT deficiency that likely contributed to the thrombotic phenotype given that another family member with a homozygous HCII deficiency had no history of VTE (91). It is possible that additional thrombophilias are required to reveal the thrombotic phenotype of HCII deficiency. Indeed, compound heterozygous mutations in HCII and either factor V or protein C have been reported to result in thrombotic manifestations (93).

Anecdotal case series-based evidence has led to further systematic studies aimed at identifying a formal association between HCII deficiency and risk of thrombosis. In a study of 277 individuals with unexplained VTE 3 cases of HCII deficiency were identified (94). However, it was determined that HCII deficiency was equally prevalent in a healthy control population and thus unlikely to be a major driver of thrombotic risk (94). In a study of 583 individuals with anticoagulant protein deficiencies no association between HCII deficiency and VTE was observed (95). It is important to note, however, that this study included only 6 individuals with HCII deficiency, of which 4 had suffered a thrombotic event (95). Owing to the small number of HCII deficient individuals the study was likely not sufficiently powered to determine an association between HCII deficiency and VTE risk (95). Taken together, the available evidence does not support an association between HCII deficiency and thrombotic events. This is in contrast to the strong association between AT deficiency and VTE, suggesting that HCII may be a less important anticoagulant compared to AT. However, given the apparent rarity of HCII deficiency additional larger scale studies may provide further insights.

The presence of advanced atherosclerotic disease in several patients with HCII deficiency has led to the suggestion that HCII may have additional vascular protective effects (90, 96). Despite the lack of an association between plasma HCII and thrombosis, significant associations between plasma levels of HCII and atherosclerotic disease have been reported (Table 2) (97). In one study of 306 elderly individuals higher plasma levels of HCII were associated with reduced carotid artery plaque thickness (98). In a second study of 134 sequential patients undergoing percutaneous coronary intervention high plasma levels of HCII were associated with a reduced rate of in-stent restenosis (99). Complementary findings were made in a similar study of 63 patients undergoing percutaneous interventions for peripheral artery disease where high plasma levels of HCII were associated with reduced restenosis (100).

### Preclinical Phenotypes

HCII deficient mice have been used to study the anticoagulant effect of endogenous HCII (101). On first description HCII deficient mice were generated at the expected frequency demonstrating normal development and survival (102). However, in a subsequent study strain dependent embryonic lethality of homozygous HCII deficient mice was observed (103). In a carotid artery rose bengal induced thrombosis model HCII deficient mice were found to have significantly shortened occlusion times. Importantly, administration of dermatan sulfate was found to prolong occlusion times in wildtype mice but not HCII deficient mice (104). Moreover, in contrast to wildtype HCII protein or HCII variant protein with reduced heparin binding (K173Q), variant HCII protein with reduced affinity for dermatan sulfate (R189H) was not able to normalize occlusion times (105). These findings suggest that dermatan sulfate is an important activator of HCII *in vivo*. Consistent with the anticoagulant activity of HCII, administration of exogenous purified HCII was found to significantly prolong occlusion times in a rat femoral artery rose bengal induced thrombosis model (106).

In addition to anticoagulant functions an important vascular protective function of HCII has been described (101). When crossed onto a ApoE deficient background mice with heterozygous or homozygous HCII deficiency demonstrated enhanced atherosclerotic lesion development (107, 108). A number of studies have also shown that heterozygous or homozygous HCII deficient mice had increased intimal hyperplasia in arteries subject to cuff or wire-based injury (103, 107, 108). The mechanism by which HCII deficiency promotes these pathologic processes has yet to be determined. Taken together these studies indicate that HCII has important anticoagulant and vascular protective activity in preclinical models of atherothrombotic disease (101). Further preclinical studies are warranted to evaluate if endogenous HCII plays a role in venous thrombosis.

### Summary

HCII is a potent GAG-enhanced inhibitor of thrombin. Available clinical evidence is limited and does not support an association between HCII deficiency and VTE. However, in preclinical studies endogenous and exogenous HCII inhibited arterial thrombosis. Critically high plasma levels of HCII have been associated with reduced atherosclerosis, a finding that has been complemented by preclinical studies.

## Protein Z Dependent Protease Inhibitor

### Biological Properties

PZPI is a 72 kDa serine protease inhibitor encoded by the *SERPINA10* gene. PZPI is present in plasma at a concentration of approximately 5 μg/ml (109). PZPI, in complex with the vitamin K-dependent cofactor protein Z (PZ), functions as a selective inhibitor of FXa, FIXa, and FXIa (Table 1) (110, 111). Lipid membrane surface and heparin have been identified as additional co-factors that enhance the inhibitory activity of PZPI toward FXa and FXIa (111–114). Although it was initially thought that FXa bound to FVa in the prothrombinase complex was protected from PZPI mediated inhibition, recent work has demonstrated that FXa is effectively targeted for inhibition by PZ bound PZPI (115). Whereas binding of PZ to PZPI was found to markedly enhance inhibitory activity toward FXa, PZPI efficiently inhibits FIXa in the absence of PZ (116). In fact, PZ binding has been found to reduce the efficiency with which PZPI inhibits FXIa (117).

### Clinical Phenotypes

Mutations in the PZPI gene *SERPINA10* have been associated with an increased risk of VTE (Table 2). In one study, sequencing of the *SERPINA10* gene revealed a significantly increased prevalence of loss of function PZPI variants (R67X, W303X) in 250 VTE patients compared to a cohort of 250 control individuals (118). A subsequent study of 1,018 VTE patients and 1,018 healthy controls confirmed that the presence of the R67X variant was associated with a significantly increased risk of VTE (119). Assessment of an additional set of *SERPINA10* mutations resulting in nonsense and loss of function PZPI variants (R88X, W324X, Q384R, F145L) in a cohort of 550 VTE patients and 600 healthy controls found that these variants were significantly more prevalent in VTE patients (120). It is important to note that the association between PZPI loss of function mutations and VTE risk was not confirmed in other similarly sized independent studies (121–124). It is possible that the association only holds for specific populations. The conflicting reports of the association between PZPI and thrombosis mirror that reported for the cofactor PZ in the setting of ischemic stroke with both positive and negative studies reported (125–128). Further population-based studies are required to more conclusively determine if an association between PZPI and VTE exists.

### Preclinical Phenotypes

Preclinical studies have demonstrated an important role for PZPI and the cofactor PZ as regulators of coagulation. Mice deficient for PZ were generated at the expected frequency. However, when PZ deficient mice were crossed onto a prothrombotic homozygous FV Leiden background complete postnatal lethality was observed (129). A high proportion of FV Leiden embryos deficient for PZ had evidence of hemorrhage, which was thought to be secondary to a consumptive coagulopathy, and intravascular deposition of fibrin (129). Similarly, PZPI deficient mice were generated at the expected frequency but when crossed onto a homozygous FV Leiden background demonstrated complete postnatal lethality (130). When crossed to generate FV Leiden heterozygotes, deficiency for PZPI, but not PZ, was found to result in embryonic lethality at mid to late gestation (130). The stronger phenotype associated with PZPI deficiency in this setting suggests that PZPI may be an important regulator of FIXa and FXIa during embryonic development (130). Interestingly, equivalent phenotypes have been observed in PZPI deficient and PZ deficient mice in the absence of an additional procoagulant phenotype. Both PZPI and PZ deficient mice demonstrated significantly reduced survival in a collagen epinephrine model of pulmonary embolism. Further, both PZPI and PZ deficient mice demonstrated increased occlusion in a carotid artery ferric chloride model (130). The equivalent phenotype associated with PZPI and PZ deficiency alone suggests that an additional procoagulant stimulus is required to reveal phenotypic differences between the SERPIN and cofactor (130). Modulating the anticoagulant activity of PZPI and PZ has also been evaluated as a potential therapy for hemophilia. Deficiency for either PZPI or PZ was found to significantly reduce bleeding times in FVIII deficient mice and supported enhanced plasma thrombin generation (131).

### Summary

PZPI is a selective inhibitor of FXa, FIXa, and FXIa. Clinical studies provide evidence of a potential association between loss of function PZPI variants and VTE risk. Complementary preclinical studies have shown that loss of PZPI or its cofactor PZ enhances arterial thrombus formation in mouse models. PZPI is a potential target for novel hemophilia therapies given its function as an inhibitor of FIXa. Indeed, PZPI deficiency improved the hemostatic response in a mouse model of hemophilia A.

## C1-Inhibitor

### Biological Properties

C1INH is a 105 kDa serine protease inhibitor encoded by the *SERPING1* gene. C1INH is present in plasma at a concentration of 200 μg/ml with a circulating half-life of approximately 3 days (132). C1INH is also present in platelet alpha granules and is released upon platelet activation (133). C1INH serves as the major endogenous inhibitor of FXIIa and plasma kallikrein (Table 1) (134–137). C1INH is also an effective inhibitor of FXIa (138). As the name indicates, C1INH was first identified for the ability to inhibit the first component of complement (139). C1INH also inhibits a host of other proteases, including plasmin, tissue plasminogen activator, mannan-binding lectin serine protease 1 and mannan-binding lectin serine protease 2 (140–142). Importantly GAGs have been found to selectively enhance the activity of C1INH toward FXIa (37, 143). However, GAGs were found to decrease inhibitory activity of C1INH toward FXIIa (37, 144). More recently the polyanion polyphosphate has been found to enhance the inhibitory activity of C1INH toward the first component of complement (145). The effect of polyphosphate on inhibition of coagulation proteases by C1INH has yet to be determined. Unlike other anticoagulant SERPINs, C1INH undergoes extensive glycosylation resulting in a markedly increased apparent molecular weight. C1INH contains 3 C-terminal and 3 N-terminal N-linked glycosylation sites and up to 26 O-linked N-terminal glycosylation sites (146). The impact of glycosylation on C1INH function remains unclear with conflicting reports on the contribution of glycosylation to circulating half-life and inhibitory efficacy of C1INH (147–150).

### Clinical Phenotypes

C1INH deficiency in humans results in a condition called hereditary angioedema (HAE), a life-threatening syndrome triggered by episodes of bradykinin induced swelling that can lead to asphyxiation (151). Mutations in the *SERPING1* gene can result in reduced expression of C1INH, leading to type I HAE, or expression of C1INH with reduced function, leading to type II HAE (151). Interestingly, the vast majority of patients presenting with HAE are heterozygous for these mutations indicating an autosomal dominant pattern of inheritance (152). HAE patients with heterozygous mutations in the C1INH gene typically present with plasma levels of C1INH antigen or activity markedly lower than the expected 50% (153). This may be due to the fact that variant C1INH proteins can form intracellular aggregates with wildtype protein leading to reduced secretion (154). To date over 500 disease-causing SERPING1 variants have been reported (152).

A number of treatments have been used to manage attacks in patients with HAE (151). C1INH products have been developed to serve as replacement therapies and include human purified plasma-derived and recombinant C1INH protein preparations (151). Plasma-derived C1INH products have elimination half-lives of 2–5 days depending on the route of administration (132). Additional therapies including the bradykinin receptor inhibitor icatibant and the kallikrein inhibitors ecallantide and berotralstat have also been developed (151). These therapies function by either inhibiting bradykinin generation or inhibiting activation of the bradykinin receptor that drives swelling in patients with HAE.

Given that the primary phenotype of patients with HAE is related to excess activation of FXIIa and kallikrein mediated bradykinin generation, efforts have been made to evaluate the effect of C1INH deficiency on the contact system (155). Levels of plasma kallikrein and FXIIa activity were markedly elevated in patients with HAE when compared to controls (156). Similarly, levels of FXIIa and cleaved kininogen were markedly higher in patients with HAE during acute attacks compared to when in remission (157).

HAE patients have been reported to have elevated plasma levels of markers of activation of coagulation and fibrin degradation, including prothrombin fragment F1+2, thrombin-antithrombin (TAT) complexes and D-Dimer compared to healthy controls (157–159). Further, these markers are elevated in HAE patients during acute attacks compared to when in remission (157–159). Activated partial thromboplastin times were significantly shorter in patients with HAE when compared to controls and significantly shorter in HAE patients during attacks than during remission (159). Plasma from patients with HAE was also found to support a modest but significant increase in TF-initiated thrombin generation when compared to controls (160). This study was conducted in the absence of a contact pathway inhibitor, such as corn trypsin inhibitor, and as such this phenotype could be attributed to residual activation of the contact pathway (160).

The relationship between C1INH deficiency and thrombosis has been less clear. Initial observations suggested that treatment of HAE patients with C1INH was associated with an increased risk of thrombosis (161). However, a large-scale registry study did not observe an association between C1INH treatment and thrombotic events (162). In a recent study HAE was associated with an increased risk of ATE (odds ratio 6.7) and VTE (odds ratio 4.2) (BRSK01,). Further, in a retrospective study treatment of HAE patients with C1INH resulted in a 10-fold reduction in the incidence of VTE on long-term follow-up compared to untreated patients (164). In a small plasma proteomics-based biomarker discovery study an association between plasma levels of C1INH and future risk of VTE was observed (Table 2) (165). In this study, low plasma levels of C1INH were found to negatively associate with future risk of VTE (165). This finding is consistent with the anticoagulant activity of C1INH and suggests that plasma levels of C1INH modulate VTE risk in the general population. Epidemiological studies have also demonstrated that elevated plasma levels of complement factor C3 and C5 are associated with an increased risk of VTE (166, 167). It is possible that the anti-complement activities of C1INH could contribute to the observed association with VTE. Further work is required to investigate this association and the contributing mechanisms.

It is interesting to note that in addition to possessing anti-complement and anti-coagulant properties, C1INH is also an acute phase reactant with anti-inflammatory properties (168). Indeed, C1INH administration reduced plasma levels of inflammatory cytokines in a clinical endotoxemia model (169). Further, C1INH administration reduced plasma levels of inflammatory cytokines and mortality in patients with sepsis (170).

### Preclinical Phenotypes

Mice deficient for C1INH have been generated to model HAE (171). C1INH deficient mice developed normally and were present at the expected frequency (171). As observed in patients with HAE, mice with a heterozygous deficiency for C1INH had markedly lower plasma C1INH levels than the expected 50% (171). Consistent with the clinical phenotype, bradykinin-mediated vascular permeability was increased in C1INH deficient mice upon challenge and could be corrected by C1INH replacement or inhibition of bradykinin receptor signaling (171).

The anticoagulant activity of exogenous C1INH has been evaluated in the preclinical setting. Administration of human purified C1INH to rabbits significantly reduced intrinsic pathway-initiated thrombin generation and prolonged the activated partial thromboplastin time (172). In a rabbit femoral artery ferric chloride thrombosis model administration of human C1INH significantly reduced vessel occlusion (172). This study demonstrates that exogenous C1INH is effective in limiting activation of coagulation and arterial thrombosis. Additional studies are required to evaluate the role of C1INH in other thrombotic pathologies.

### Summary

C1INH is a major endogenous inhibitor of intrinsic pathway factors PKa, FXIIa, and FXIa. A congenital deficiency in C1INH, causing HAE, has been shown to result in increased plasma levels of markers of activation of coagulation. Epidemiological evidence has indicated that low plasma levels of C1INH are associated with an increased risk of VTE. Preclinical studies indicated that exogenous C1INH is able to effectively suppress intrinsic pathway mediated activation of coagulation and arterial thrombosis.

## Protease Nexin 1

### Biological Properties

PN1 is a 50 kDa protein encoded by the *SERPINE2* gene and functions as a broad serine protease inhibitor. PN1 is a potent inhibitor of the coagulation proteases thrombin and FXIa, and to a lesser extent FXa (Table 1) (173, 174). PN1 also shows inhibitory activity toward trypsin, urokinase plasminogen activator and plasmin (173). As with other anticoagulant SERPINs the activity of PN1 toward target proteases is significantly enhanced by heparin (173). However, unlike the majority of other anticoagulant SERPINs, PN1 is not expressed by the liver and is not present at detectable levels in plasma (175). Instead, PN1 is expressed in a number of other tissues, including the brain, heart, spleen, kidney and lung (176). PN1 is also present in platelets and monocytes (177). PN1 is stored in platelet alpha granules that may serve as a labile pool of this SERPIN (178). It is likely that release of PN1 from activated platelets accumulating at sites of vascular injury results in high localized levels of this SERPIN. Such a regulated mechanism would be distinct from that of other anticoagulant SERPINs that are present at high levels in plasma.

### Clinical Studies

The *SERPINE2* gene was identified as a candidate susceptibility gene for chronic obstructive pulmonary disease (179). The association between *SERPINE2* gene polymorphisms and chronic obstructive pulmonary disease was confirmed in a subsequent study (180). Additional studies indicate that *SERPINE2* polymorphisms may be associated with a wider spectrum of lung diseases, including asthma and emphysema (181–183). To date, *SERPINE2* polymorphisms have not been associated with thrombotic pathologies.

### Preclinical Studies

PN1 deficient mice have been developed as a tool to study the biological function of this SERPIN (184). PN1 deficient mice were viable and developed normally with no overt phenotype (184). Subsequent studies demonstrated that mice deficient for PN1 had markedly enhanced thrombus formation in both mesenteric venule and arteriole ferric chloride injury models (178, 185). Platelet PN1 was found to inhibit thrombin activity with platelet-rich plasma from PN1 deficient mice supporting increased thrombin generation (178). Interestingly, platelets from PN1 deficient mice also demonstrated enhanced P-selectin exposure and aggregation in response to thrombin (178). These findings suggest that the platelet is an important source of PN1 and confers anticoagulant activity through the ability to inhibit thrombin (185). Consistent with the clinically observed lung phenotype, PN1 deficient mice have reduced survival in a bleomycin induced lung injury model (186). Loss of PN1 led to increased inflammation and activation of coagulation in the lung (186). Bone marrow chimeras demonstrated that this phenotype was driven by loss of PN1 in hematopoietic cells and could be reversed by inhibition of thrombin or PAR4 activation (186).

The effect of PN1 on hemostatic processes has also been investigated. Plasma from FVIII deficient mice also deficient for PN1 or treated with an anti-PN1 antibody supported enhanced thrombin generation (187). FVIII deficient mice also deficient for PN1 demonstrated markedly reduced bleeding in a tail amputation model compared with controls (187). Further, translating these findings anti-PN1 single domain antibodies have been developed (188). The anti-PN1 antibodies restored thrombin activity in purified and plasma-based systems (188). The procoagulant effect of anti-PN1 antibodies could find utility as a novel hemostatic therapy for patients with hemophilia.

In addition to inhibiting procoagulants PN1 also inhibits fibrinolytic enzymes. *Ex vivo*, tPA initiated clot lysis was enhanced in plasma from PN1 deficient mice. *In vivo*, thrombi formed in PN1 deficient mice were found to be more susceptible to tPA induced lysis (189). This suggests that PN1 has important anticoagulant and antifibrinolytic functions. While PN1 may inhibit thrombus formation, thrombi formed in the presence of PN1 may be more resistant to lysis.

### Summary

PN1 functions as an inhibitor of thrombin, FXa and FXIa. Although PN1 variants are associated with pulmonary pathologies, their effects on thrombotic and hemostatic disorders have not been reported. In preclinical studies, PN1 deficiency was found to enhance thrombus formation in mice. Additional studies indicate that PN1 may be a suitable target for novel hemophilia therapies with inhibition of PN1 found to normalize hemostasis in a mouse model.

## Protein C Inhibitor

### Biological Properties

PCI is a 46 kDa serine protease inhibitor encoded by the *SERPINA5* gene and is present in plasma at a concentration of roughly 5 μg/ml (190). PCI is a potent inhibitor of the anticoagulant aPC conferring PCI with procoagulant activity, unlike the other SERPINs reviewed here (190). However, in addition to aPC, PCI also inhibits several coagulation proteases, including thrombin, FXa, FXIa, and plasma kallikrein providing PCI with anticoagulant activity (Table 1) (191). As with other SERPINs the activity of PCI against coagulation proteases is enhanced by heparin (191). In addition, the activity of PCI against thrombin is markedly enhanced by thrombomodulin (192).

### Clinical Studies

To date no congenital deficiency for PCI has been described. Early studies postulated that PCI deficiency may underpin combined FV and FVIII deficiency as plasma PCI activity was undetectable in patients with this deficiency (193). Mechanistically, an inability to inhibit aPC could lead to a constitutive reduction in FVa and FVIIIa activity. Subsequent studies revealed that individuals with combined FV and FVIII deficiency had normal levels of functional PCI (194). Involvement of PCI in the combined deficiency was more conclusively excluded when the locus for the SERPINA5 gene was mapped on chromosome 14 with the genetic defect associated with FV and FVIII deficiency mapped to chromosome 18 (195, 196). Although there have been no reports on individuals with congenital PCI deficiency, available evidence from the whole exome sequencing database gnomAD indicates that predicted loss of function variants are present at the expected frequency (197).

### Preclinical Studies

Studies of PCI in mice are complicated by the fact that the pattern of tissue expression in this species differs compared to humans. Whereas PCI is expressed in the liver in humans expression is largely absent in the liver of mice (198). Given that the liver is a critical organ for release of proteins into the blood the lack of expression in this organ in mice likely explains the observed absence of PCI in mouse plasma (199). These species differences notwithstanding, PCI deficient mice have been generated (199). PCI deficient mice were generated at the expected frequency and did not demonstrate any gross hemostatic defects. However, male PCI deficient mice were found to be infertile (199). PCI was found to be present at very high levels in human seminal fluid suggesting an important role in male fertility (200). Further studies demonstrated impaired spermatogenesis in PCI deficiency mice (201). To study the role of plasma PCI in the mouse a transgenic approach was used in which human PCI was expressed in the mouse liver (198). Transgenic overexpression of PCI resulted in mouse plasma levels of human PCI roughly double that observed normal human plasma (198). As expected, human PCI transgenic mouse plasma was able to effectively inhibit exogenous human aPC (198). However, in an LPS endotoxemia model no difference in survival was observed between human PCI transgenic mice and controls (198). An additional transgenic mouse expressing human PCI under the control of the human promoter resulted in plasma levels of PCI approximately four times that found in human plasma (202). In these mice expression of human PCI resulted in shortened activated partial thromboplastin plasma clotting times (202). Interestingly, in an LPS endotoxemia model these human PCI transgenic mice demonstrated an enhanced prolongation of activated partial thromboplastin times and an enhanced reduction in plasma fibrinogen compared to controls (202). The high plasma levels of PCI in these mice likely inhibited endogenous mouse aPC leading to a consumptive coagulopathy. The findings with human PCI transgenic mice indicate that the predominant effect of PCI is as a procoagulant.

### Summary

Despite being an inhibitor of multiple coagulation proteases the potent activity of PCI toward aPC confers this SERPIN with net procoagulant activity. No clinical association between PCI and bleeding or thrombosis has been established. Preclinical studies indicate that PCI potently enhances activation of coagulation induced by endotoxemia.

## Alpha 1 Antitrypsin

### Biological Properties

A1AT is a 53 kDA serine protease inhibitor encoded by the *SERPINA1* gene. A1AT circulates in plasma at a concentration of approximately 1.2 mg/ml with a half-life of 4–5 days. Although A1AT is primarily expressed in the liver it is also produced in monocytes and macrophages owing to the presence of an alternative promoter (203). A1AT serves as the major endogenous inhibitor of neutrophil elastase (204). A1AT also functions as a potent inhibitor of other neutrophil related proteases, including proteinase 3 and cathepsin G (205, 206). A1AT demonstrates weak inhibitory activity against FXa and FXIa (Table 1) (37, 207). Although GAGs enhance the inhibitory activity of a number of SERPINs in the case of A1AT the presence of heparin has been found to markedly reduce inhibitory activity (37, 208).

### Clinical Phenotypes

Congenital A1AT deficiency is a common genetic disease (209). The vast majority of severe A1AT deficiencies result from homozygosity for a single variant that causes an amino acid substitution (Q342K) frequently referred to as the Z allele (209). The Z allele is particularly prevalent in individuals of European descent (heterozygous, 1:25: homozygous 1:2,000) (209). Variant Z allele protein polymerizes in the endoplasmic reticulum resulting in reduced secretion (210). Another single acid substitution (Q264V) referred to as the S allele is less common and results in more modest A1AT deficiency (211). Despite a preponderance for the Z and S alleles in individuals with A1AT deficiency to date more than 120 variants have been described causing some degree of A1AT deficiency (212). Individuals with A1AT deficiency are at increased risk of emphysema and chronic obstructive pulmonary disease (213, 214). Indeed, 5% of all chronic obstructive pulmonary disease diagnoses may be attributable to A1AT deficiency (215). Individuals with A1AT deficiency were also found to be at increased risk of liver disease (213, 216). Variant Z allele A1AT has been shown to fold incorrectly forming toxic aggregates in hepatocytes that function as a driver of liver disease in this patient population (217). Human purified plasma-derived A1AT augmentation therapies with elimination half-lives of 2–3 days have been developed as effective treatments for patients with A1AT deficiency associated chronic obstructive pulmonary disease (218).

More recently, it was found that individuals with A1AT deficiency are at increased risk of VTE (Table 2). In one study, individuals with severe A1AT deficiency, caused by homozygosity for the Z allele, had a significantly increased risk of VTE compared to controls (hazard ratio 4.2) (219). In a second population-based study individuals homozygous for the Z allele were also found to have a significantly increased risk of VTE (hazard ratio 2.2) (220). Interestingly, severe A1AT deficiency has been associated with a reduced risk of ischemic heart disease (221). The mechanism by which loss of endogenous A1AT results in an increased risk of VTE but reduced risk of ischemic heart disease remains to be determined.

A naturally occurring A1AT variant, M358R, termed A1AT-Pittsburgh was identified as the underlying genetic cause of recurrent bleeding events in a young patient (222). Subsequent studies revealed that the M358R single amino acid substitution transformed A1AT from an inhibitor of neutrophil proteases to a broad inhibitor of coagulation associated proteases, including thrombin, aPC, plasmin, FXIa, FXa, plasma kallikrein, and FXIIa (223–225). The A1AT-Pittsburgh variant is very rare with only 2 pedigrees reported to date and is associated with a variable bleeding phenotype (222, 226).

A1AT is an acute phase reactant reported to have important anti-inflammatory and immunomodulatory functions. A1AT deficiency is associated with the inflammatory condition rheumatoid arthritis (227, 228). The anti-inflammatory and anti-viral properties of A1AT products are currently being evaluated in the setting of COVID-19 (229).

### Preclinical Phenotypes

A1AT deficiency has proven challenging to model in mice owing to the presence of fives *SERPINA1* paralogs. Early efforts deleting a single *SERPINA1* paralog resulted in unexpected embryonic lethality (230, 231). This is not consistent with the observed phenotype of humans with a severe A1AT deficiency and suggested that murine A1AT may have gained additional developmental functions in the mouse. More recently, however, a complete A1AT deficient mouse has been developed lacking all five of the murine *SERPINA1* paralogs (232). These A1AT deficient mice demonstrated normal survival (232). Consistent with the reported role of A1AT deficiency in lung disease, aged deficient mice demonstrated spontaneous emphysema with evidence of increased neutrophil, monocyte and lymphocyte counts in bronchial alveolar lavage fluid (232). Further studies are required to determine if these deficient mice can model the prothrombotic phenotype observed in humans (232).

### Summary

The primary activity of A1AT is as a potent inhibitor of neutrophil-derived proteases. A1AT possesses relatively weak anticoagulant activity. Despite this weak anticoagulant activity recent clinical studies have revealed that patients with A1AT deficiency are at increased risk of VTE. The mechanism by which endogenous A1AT prevents VTE remains to be elucidated.

## Engineered Serine Protease Inhibitor

Generation of SERPIN variant proteins was initially used as a powerful tool to decipher what components of a given SERPIN’s structure contributed to the observed biological activity. For example, generation of variants has facilitated the identification of GAG binding regions and provided mechanistic insights into how these molecules enhance SERPIN activity. However, based on the important contribution of anticoagulant SERPINs to hemostatic and thrombotic processes considerable efforts have also been made to engineer variants with altered biological activities and properties (19, 233, 234). Engineered variant SERPIN proteins are typically designed to either alter substrate selectivity or improve biological stability. The overarching goal of these efforts is to generate novel engineered SERPINs that possess attractive biochemical and biophysical properties for potential use as novel therapeutic agents.

### Altered Substrate Selectivity

Significant efforts have been made to alter and refine the selectivity of a number of anticoagulant SERPINs. This has primarily been achieved by substitution of residues in the RCL that serves as an important determinant of selectivity. Initial efforts focused on generating chimeric SERPINs that substituted partial RCL sequence of one SERPIN into another. Using this approach chimeric SERPINs have been made that swap portions of the RCL of AT into A1AT and plasminogen activator inhibitor 1 (235–237). While in both cases insertion of the AT RCL conferred thrombin inhibitory capacity the second order rate constants were markedly lower than that of wildtype AT. This reinforces that although the RCL sequence is an important determinant of substrate selectivity other regions play a role.

Further efforts have focused on single or multiple amino acid substitutions of the RCL in a given SERPIN. A1AT has been the focus of concerted efforts likely owing to the presence of the naturally occurring A1AT-Pittsburgh variant, M358R, that demonstrated the profound effects that a single amino acid substitution could have on substrate selectivity (223–225). An additional amino acid substitution at the preceding P2 residue (^357^AR^358^) further modified the activity of the A1AT-Pittsburgh variant conferring increased activity toward plasma kallikrein (238). This substitution was found to protect mice from bradykinin induced hypertension (238). Greater selectivity of A1AT toward plasma kallikrein was achieved by substituting the P3 and P2 residues in the A1AT-Pittsburgh variant (^356^PFR^358^) (239). Two additional A1AT-Pittsburgh based variants have been developed (^355^SLLRV^359^ and ^355^SMTRV^359^) that demonstrate improved activity against plasma kallikrein and reduced activity against both thrombin and aPC (240). These variants were effective in reducing bradykinin induced edema and arterial thrombosis in mouse models (240). To improve the selectivity of the A1AT-Pittsburgh variant for thrombin over aPC additional A1AT-Pittsburgh based variants have been evaluated (^357^AR^358^, ^357^GR^358^, and ^358^RT^359^) but no marked difference in selectivity was observed (237).

A1AT variant design has also been informed by the crystal structure of target proteases. In one approach the A1AT variant ^357^KRK^359^ was designed to selectively inhibit aPC over thrombin due to the steric constraints of the thrombin active site (241). As predicted, the A1AT variant ^357^KRK^359^ was highly selective for aPC and significantly enhanced thrombin generation in the plasma of hemophilia A patients (241). In FVIII deficient mice the A1AT variant ^357^KRK^359^ was also found to reduce bleeding in a tail amputation model and increase platelet and fibrin accumulation at sites of vascular injury (241).

The effect of RCL amino acid substitutions in other SERPINs has also been evaluated. Systematic substitution of the P1 residue of C1INH demonstrated the importance of this amino acid to inhibitor activity and selectivity (242). The majority of C1INH P1 variants demonstrated reduced or absent activity toward C1s, plasma kallikrein, FXIIa and plasmin (242). However, one variant, R442K, demonstrated preserved activity toward C1s with reduced activity toward plasma kallikrein and FXIIa (242). A naturally occurring C1INH P2 variant, A443V, has been identified and was found to have increased specificity toward plasma kallikrein and FXIIa (243). In a systematic substitution approach for the P2 residue of C1INH the naturally occurring A443V variant was found to have increased activity toward trypsin and thrombin (244). Similarly, the P1 variant R444L was found to have increased heparin dependent activity toward thrombin (245). One additional variant of particular interest, A443T, showed markedly enhanced specificity for plasma kallikrein and FXIIa over C1s, thrombin and plasmin (244).

Larger scale systematic approaches have also been evaluated. A phage display approach has been used to identify A1AT RCL sequences with enhanced specificity for FXIa (246). In a multistage approach, fragments of the A1AT-Pittsburgh RCL sequence were mutated and screened for the ability to inhibit FXIa in a process termed biopanning. A1AT containing the FXIa optimized RCL (^346^HASTGQFLEAIPR^358^) demonstrated strong selectivity for FXIa over thrombin (246). In another approach the P4 to P4’ residues of A1AT, covering a region particularly important for substrate selectivity, were systematically substituted (247). Recombinant variant A1AT proteins containing single amino acid substitutions for every amino acid at each of these positions were generated and their activity toward target proteases evaluated (247). A platform was developed to predict the effect of multiple amino acid substitutions on substrate specificity (247). Using this platform variants with predicted potency and specificity for aPC were selected for evaluation as novel hemophilia therapies (247). A number of these predicted A1AT variants were found to be selective inhibitors of aPC. One variant, ^355^KMPRRIPA^362^, restored hemostasis more effectively in FVIII deficient mice than the previously reported A1AT ^357^KRK^359^ (247).

### Improved Half-Life

Numerous efforts have been made to improve the circulating half-life of anticoagulant SERPINS. This is due to the fact that some natural SERPINs and other recombinantly produced SERPINs have relatively short circulating half-lives.

One approach to improve the half-life of circulating SERPINs is to modify protein glycosylation in a process termed glycoengineering (248). N-linked glycosylation of natural A1AT aids in correct folding of the protein into the metastable state and extends the circulating half-life (249, 250). While purified A1AT contains normally glycosylated protein, A1AT produced recombinantly often contains sub optimally glycosylated protein. In one approach the effect of adding additional glycosylation sites to A1AT on the circulating half-life was assessed. A recombinant A1AT variant containing one additional N-linked glycosylation site (G147N/K149T) had a significantly longer circulating half-life in mice (251). Similar findings were made with a further recombinant A1AT variant containing two additional N-linked glycosylation sites (Q9N and D12N/S14T) that resulted in a significantly prolonged circulating half-life in rats (252). As an alternative approach, efforts have also been undertaken to improve glycosylation of natural sites in recombinantly produced A1AT. An optimized Chinese ovarian hamster cell line that overexpresses a human glycosylation gene produced recombinant A1AT with a glycosylation profile similar to that of purified A1AT (253). However, it remains to be determined to what extent this improves the circulating half-life of recombinant A1AT.

It should be noted that SERPIN glycosylation has effects beyond simply prolonging the circulating half-life. Wildtype AT contains four sites for N-linked glycosylation with one site demonstrating poor glycosylation. Addition of N-linked glycosylation sites has had variable effects on secretion with modifications at some positions impairing secretion while at other sites modification improved secretion (254). These differing results are likely a result of the effect of N-linked glycosylation on protein folding. Indeed, N-linked glycosylation has been shown to contribute to the efficient folding and secretion of AT (255). Additional N-linked glycosylation could also alter SERPIN activity. The potential for this to occur has been highlighted by the varying activities of the two naturally occurring forms of AT, the α and β forms. The α form of AT, that is glycosylated at all four sites, demonstrates impaired activity against thrombin compared to the β form, that is glycosylated at three sites, with the additional glycosylation being found to disrupt heparin interactions (256). This indicates that although N-linked glycosylation may improve biological half-life this modification may have unpredictable, and possibly deleterious, effects on protein secretion and function.

In an additional approach the effect of conjugating A1AT with polyethylene glycol (PEG), in a process termed PEGylation, has been evaluated. PEGylation of recombinant A1AT markedly reduced renal clearance resulting in a prolonged circulating half-life (257). PEGylation of a single amino acid residue of recombinant A1AT (C232) increased the circulating half-life in mice with longer PEG polymers having a greater effect (258). A PEGylated version of the Myxomavirus derived SERPIN Serp-1 has also been developed demonstrating improved efficacy in a mouse model of diffuse alveolar hemorrhage (259). Serp-1 is a broad acting SERPIN with anti-inflammatory and anti-fibrinolytic activities (260). In addition, however, Serp-1 also functions as a heparin dependent inhibitor of thrombin (261, 262). Serp-1 is currently being evaluated as a novel therapy for treatment of acute coronary syndrome (263). It is interesting to consider if the thrombin inhibitory activity of Serp-1 may contribute to the therapeutic effect of this SERPIN.

The generation of A1AT fusion proteins has also been explored. A1AT has been conjugated to the Fc portion of immunoglobulin (264). Conjugation of intact A1AT with the Fc portion of immunoglobulin resulted in a fusion protein that retained inhibitory activity. Although not directly determined it was inferred that this fusion protein should have a prolonged circulating half-life through interaction with Fc receptors present on immune cells (264). Importantly, A1AT-Fc was found to be more effective in preserving lung function in murine emphysema models than purified A1AT (265).

## Conclusion

Anticoagulant SERPINs serve as critical negative regulators of coagulation. Consistent with their essential anticoagulant function congenital deficiencies of specific SERPINs, such as AT and HCII, are associated with an increased risk of VTE in humans. Moreover, targeting of these anticoagulants is being explored as a novel approach to reduce bleeding in hemophilia patients. Concerted efforts have been made to develop novel therapeutic SERPINs with altered selectivity and specificity for target coagulation proteases. Such therapeutic SERPINs may find utility as novel therapies for thrombotic pathologies.

## Author Contributions

SG conducted the literature search and wrote the manuscript. NM critically reviewed and edited the manuscript. Both authors contributed to the article and approved the submitted version.

## Conflict of Interest

SG has been a consultant for CSL Behring and has received research support from CSL Behring. The remaining author declares that the research was conducted in the absence of any commercial or financial relationships that could be construed as a potential conflict of interest.

## Publisher’s Note

All claims expressed in this article are solely those of the authors and do not necessarily represent those of their affiliated organizations, or those of the publisher, the editors and the reviewers. Any product that may be evaluated in this article, or claim that may be made by its manufacturer, is not guaranteed or endorsed by the publisher.
